# Supplementary material for: Intrinsic p53 activation restricts gammaherpesvirus driven germinal center B cell expansion during latency establishment
Source: Nat Commun. 2025 Jan 22;16:951. doi: 10.1038/s41467-025-56247-5 (PMC11754798; doi:10.1038/s41467-025-56247-5)
Supplement: Supplementary file 1 — Supplementary Information [file 41467_2025_56247_MOESM1_ESM.pdf]

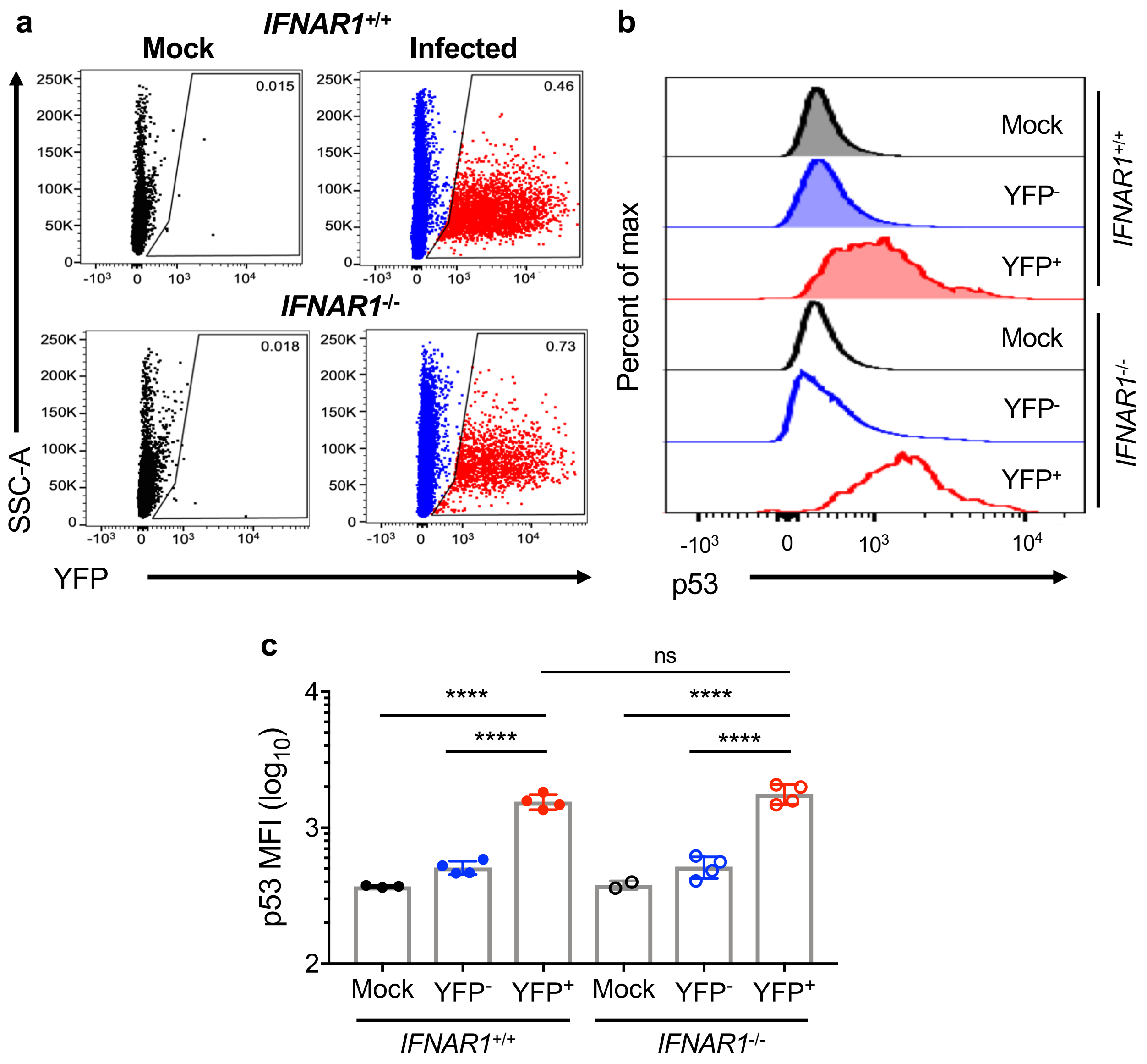

Supplementary Figure 1: MHV68 activation of p53 is interferon independent.

a-c, *IFNAR1*$^{+/+}$ or *IFNAR1*$^{-/-}$ mice were infected intranasally with MHV68 H2B-YFP (10^4 PFU). Splenocytes were harvested on day 16 post-infection. **a,** Representative flow plots for YFP expression in splenocytes. **b-c,** Flow cytometry was performed to evaluate p53 expression in mock-infected (black), MHV68$^-$ (YFP$^-$), and MHV68$^+$ (YFP$^+$) B cells. The mean fluorescence intensity of p53 is enumerated in right panel. Two-tailed Student's *t* test, **** $p<0.0001$. Each dot represents a single mouse.

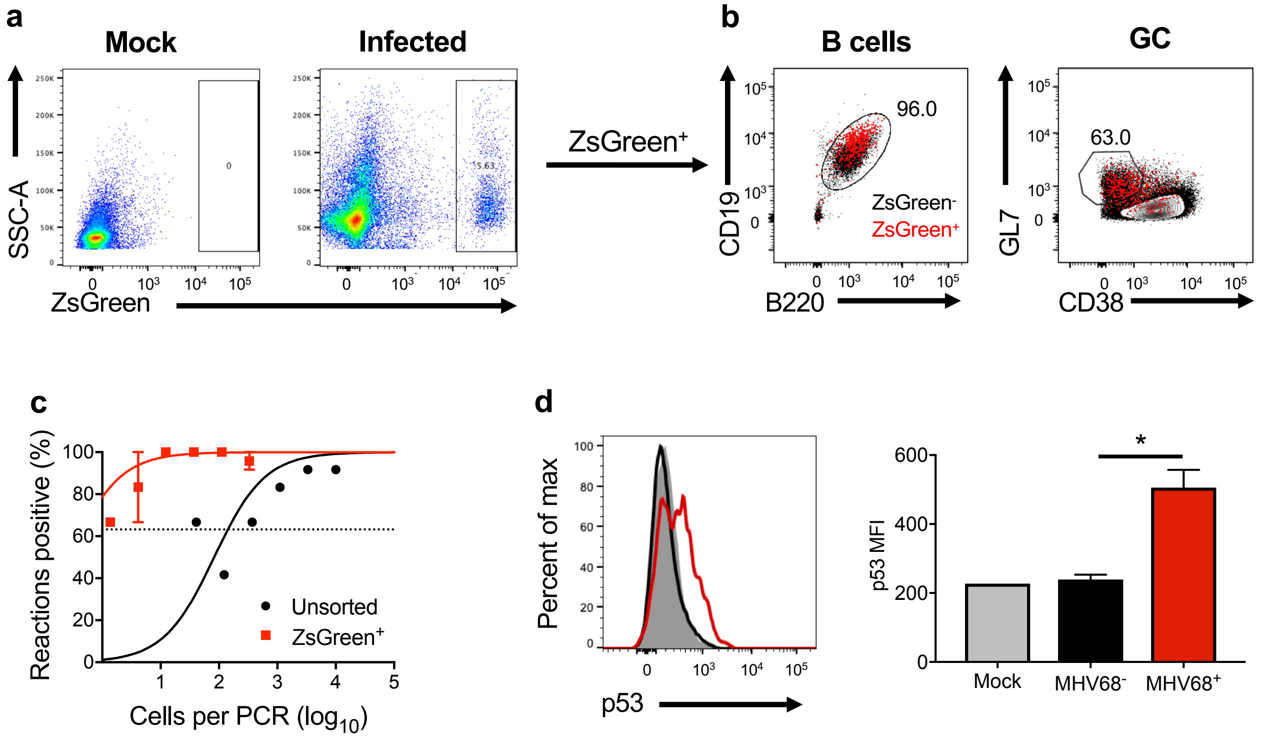

Supplementary Figure 2: Cre-loxP reporter mouse system identifies MHV68-infected B cells.
a-d, Cre-reporter mice, Ai6 (Zsgreen) or Ai14 (tdTomato), were infected intranasally with MHV68-Cre (10^4 PFU). Mediastinal lymph nodes (MLN) and splenocytes were harvested on day 16 post-infection. **a,** Representative flow plots for ZsGreen expression in splenocytes. **b,** Harvested splenocytes were stained with cell-specific markers for the indicated cell types. B cells were gated as $CD19^+/B220^+$, GC B cells as $GL7^+/CD38^{lo}$. $ZsGreen^+$ B cell populations indicated in red, $ZsGreen^-$ B cell populations indicated in black. Population frequencies apply to $ZsGreen^+$ cells. **c,** $ZsGreen^+$ cells were sorted, and limiting dilution PCR was performed to quantify the proportion of splenocytes harboring latent viral genomes. **d,** Cells from the MLN were stained with B cell markers ($B220^+/CD19^+$) and p53. Flow cytometry was performed to evaluate p53 expression in mock-infected (gray), $MHV68^-$ ($tdTomato^-$), and $MHV68^+$ ($tdTomato^+$) B cells. The mean fluorescence intensity of p53 is enumerated in right panel. Two-tailed Student's *t* test, * $p<0.05$.

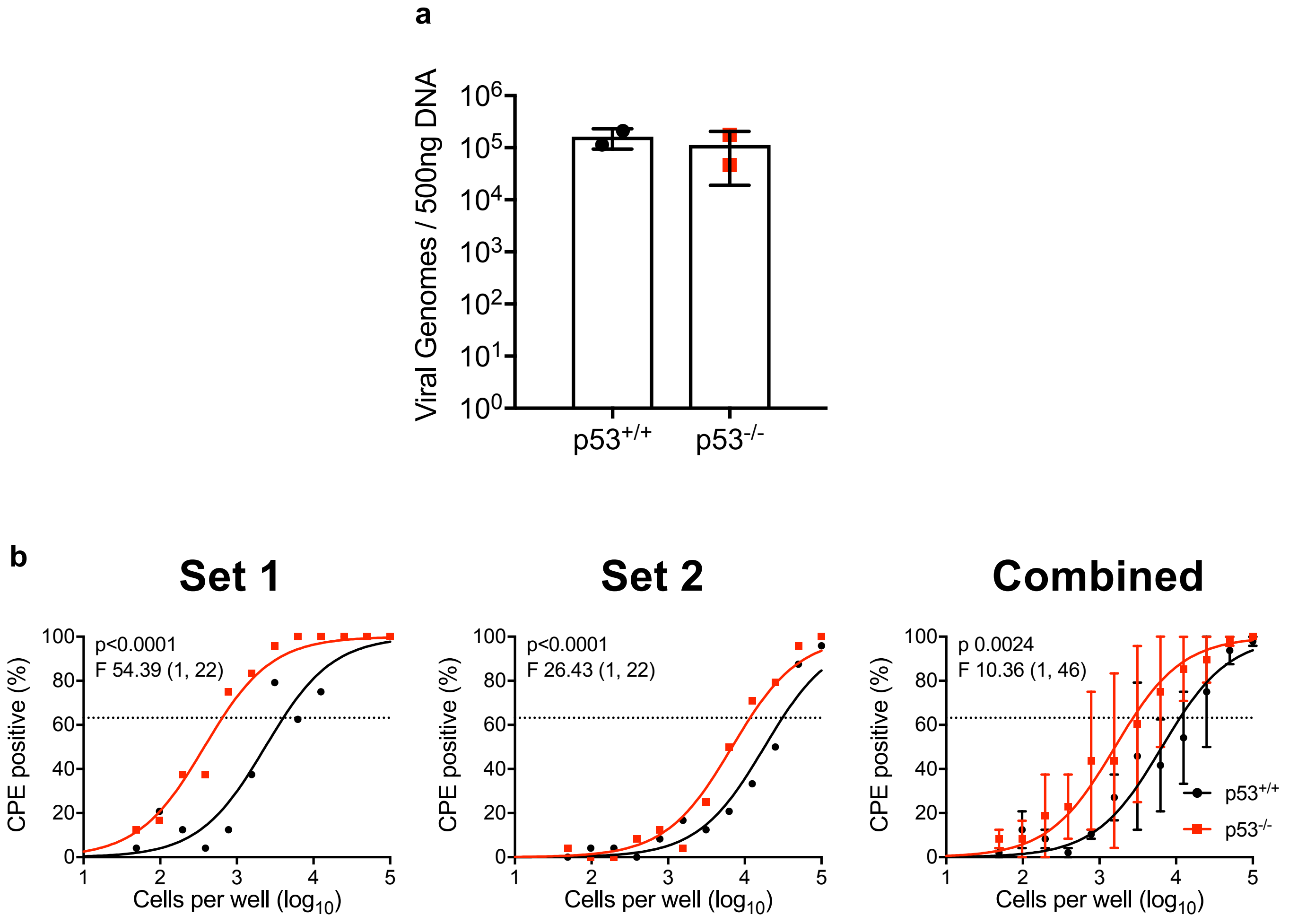

Supplementary Figure 3: p53 does not affect MHV68 acute replication and reactivation. a-b, $p53^{+/+}$ or $p53^{-/-}$ mice were intranasally inoculated with 10^4 PFU of H2B-YFP MHV68. **a,** Animals were sacrificed 7 days post-infection and DNA was isolated from lungs for quantitative PCR to detect viral genomes. Results are means of 2 independent infections with a minimum of 3 mice per group +/- SD. **b,** Animals were sacrificed 16 days after mock infection or infection of $p53^{+/+}$ or $p53^{-/-}$ mice with 10^4 PFU of H2B-YFP MHV68. Quantification of latent MHV68 reactivation efficiency from infected $p53^{-/-}$ or $p53^{+/+}$ splenocytes on day 16 post-infection. Reactivation was detected by scoring cytopathic effect in a limiting-dilution ex vivo culture. Data represent means +/- SEM for 2 independent infections with a minimum of 3 mice per group. Extra sum-of-squares F tests.

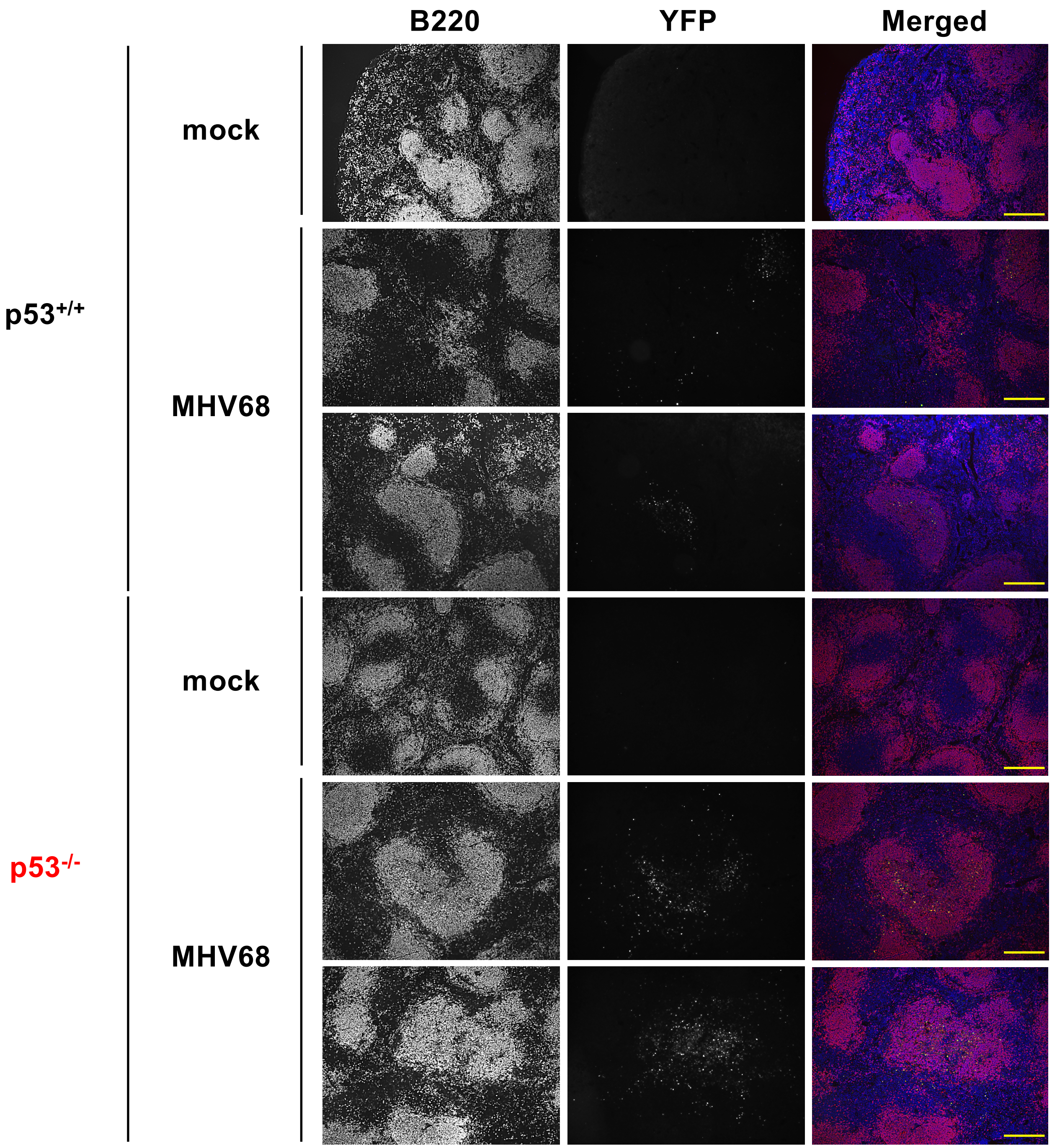

Supplementary Figure 4: Additional representative images of immunohistochemical visualization of B cells (B220+) and MHV68 infected cells (YFP+) in spleen sections from $p53^{-/-}$ or $p53^{+/+}$ mice on day 16 after infection shown in **Figure 2**. Representative images of a single follicle are shown from individual mice, n=3. Scale bars indicate 250 μM.

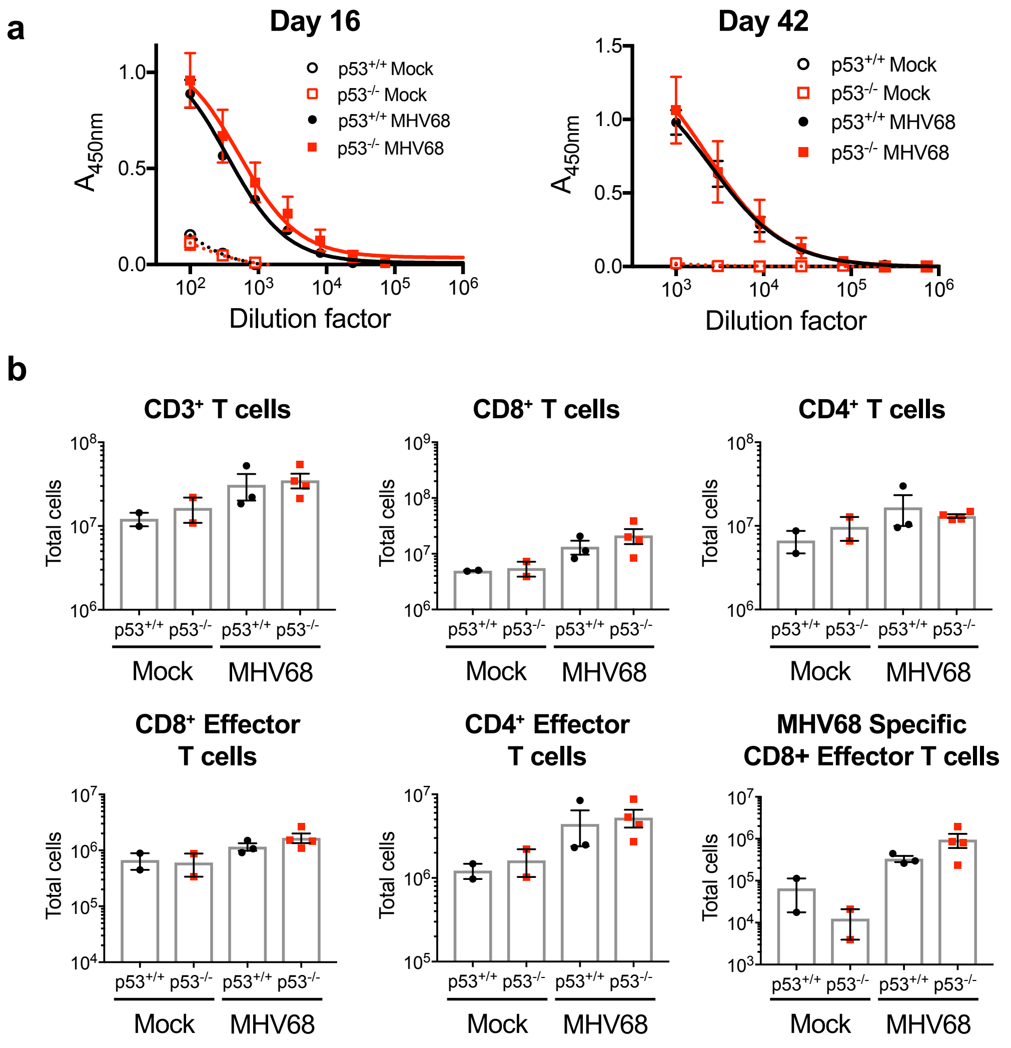

Supplementary Figure 5: p53 is not required for virus-specific adaptive immunity. a,b, $p53^{+/+}$ or $p53^{-/-}$ mice were intranasally inoculated with 10^4 PFU of H2B-YFP MHV68. **a,** Serum was collected on days 16 and 42 post-infection, corresponding to the peak and maintenance phase of viral latency, respectively. Serial dilutions were evaluated by ELISA to quantify MHV68-specific IgG. **b,** Mock-infected and MHV68-infected mice were sacrificed on day 42 post-infection. Flow cytometry was performed to quantify total T cells ($CD19^-$/$CD3^+$), $CD8^+$ T cells, $CD4^+$ T cells, effector T cells ($CD62L^-$/$CD44^+$), and MHV68-specific effector CD8 T cells that were detected by staining with an MHV68 ORF6 MHCI tetramer. Data from two independent experiments are shown. Each dot represents a single mouse. Data represent means +/- SEM. No significant differences were present by Mann-Whitney unpaired *t* test.

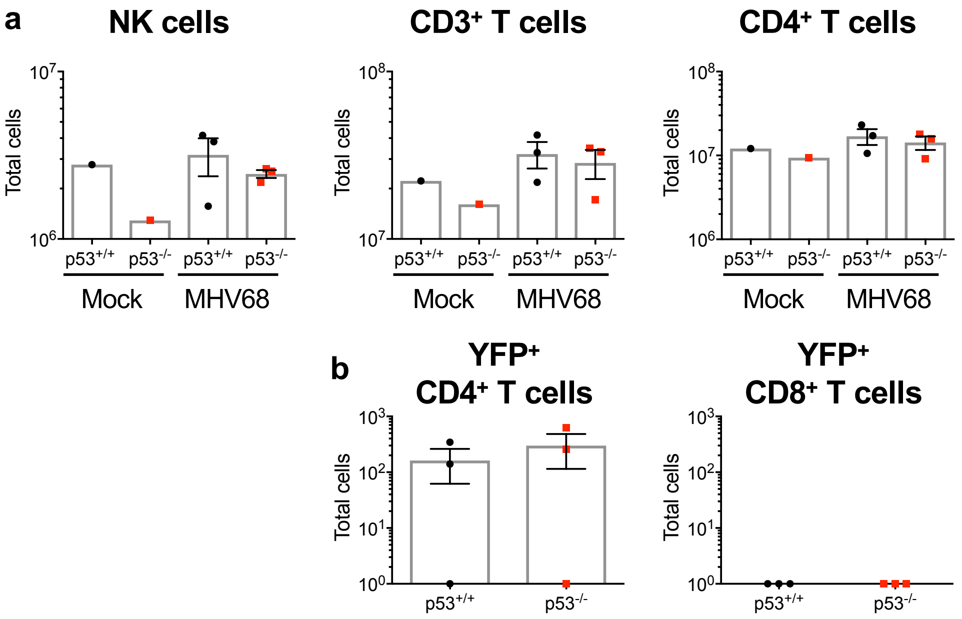

Supplementary Figure 6: p53 does not influence NK and T cell populations during MHV68 infection. **a,b,** $p53^{+/+}$ or $p53^{-/-}$ mice were infected intranasally with 10^4 PFU of H2B-YFP MHV68. Mice were sacrificed on day 16 post-infection and splenocyte populations were analyzed by flow cytometry. **a,** Total T cells were gated as $CD3^+$ and specific subsets were identified by CD8 or CD4 expression. Natural killer cells were gated as $CD3^{lo}$/$NK1.1^+$. Live cells were identified with eFluor 780 viability dye. **b,** Quantification of infected (YFP+) $CD4^+$ and $CD8^+$ T cells. Each dot represents one mouse. Data represent means +/- SEM. No significant differences were present by Mann-Whitney unpaired *t* test.

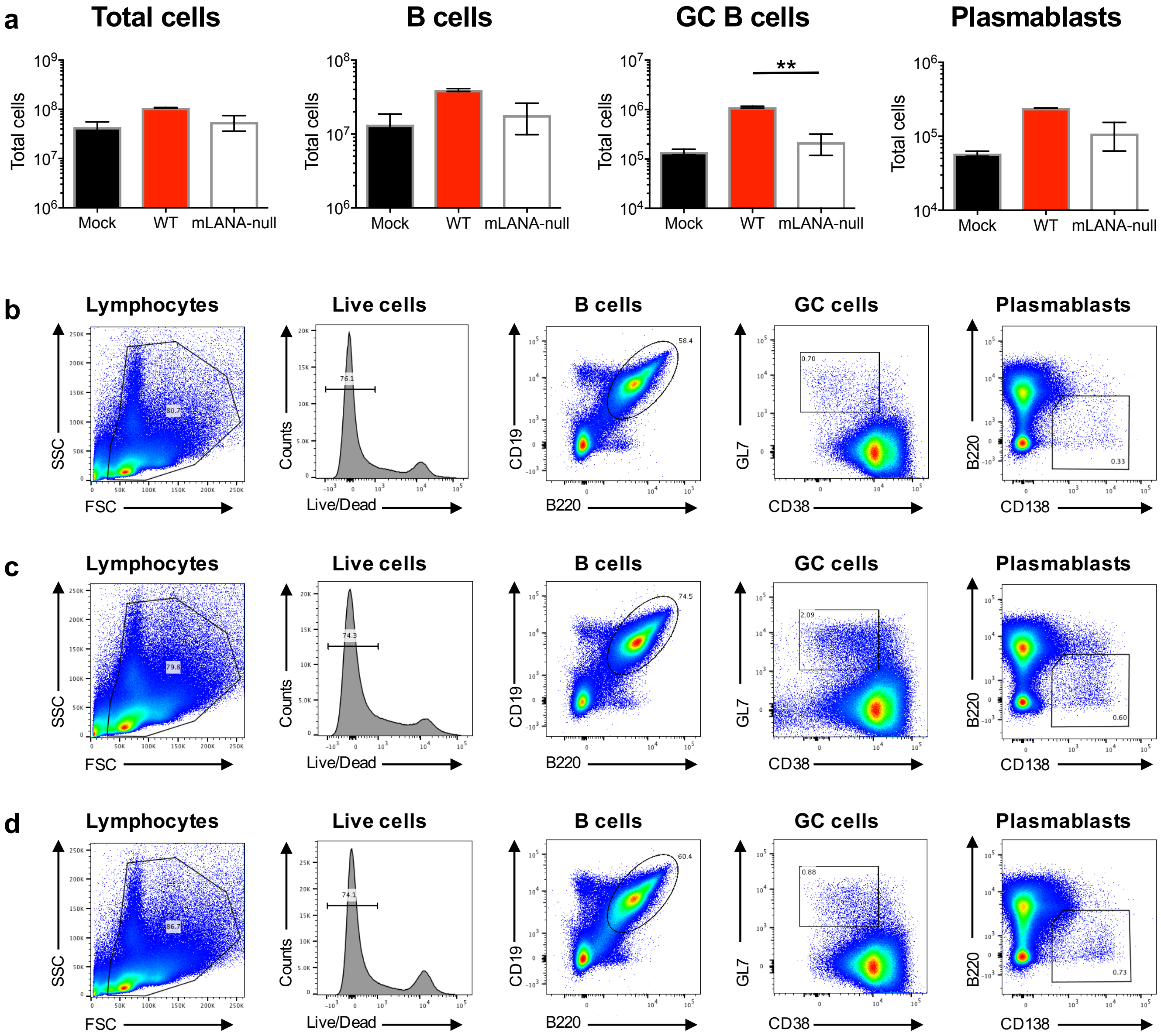

Supplementary Figure 7: Latent MHV68 infection is required for splenic B cell expansion in p53$^{-/-}$ mice. a-d, p53$^{-/-}$ mice were mock infected or infected intranasally with 10^4 PFU of H2B-YFP MHV68 or mLANA-null MHV68 (n=3). Mice were sacrificed on day 16 post-infection and splenocyte populations were defined by flow cytometry. Representative flow cytometry plots are shown for mock infection in **a**, H2B-YFP MHV68 infection in **b**, and mLANA-null MHV68 infection in **c**. B cells were gated as CD19^{+}/B220^{+}, GC B cells as GL7^{+}/CD38^{lo} subset of B cell gate, and plasmablasts as CD138^{+}/B220^{lo}. Live cells were identified with eFluor 780 viability dye. Data in **d** represent means +/- SEM. Mann-Whitney unpaired *t* test, ** $p<0.01$

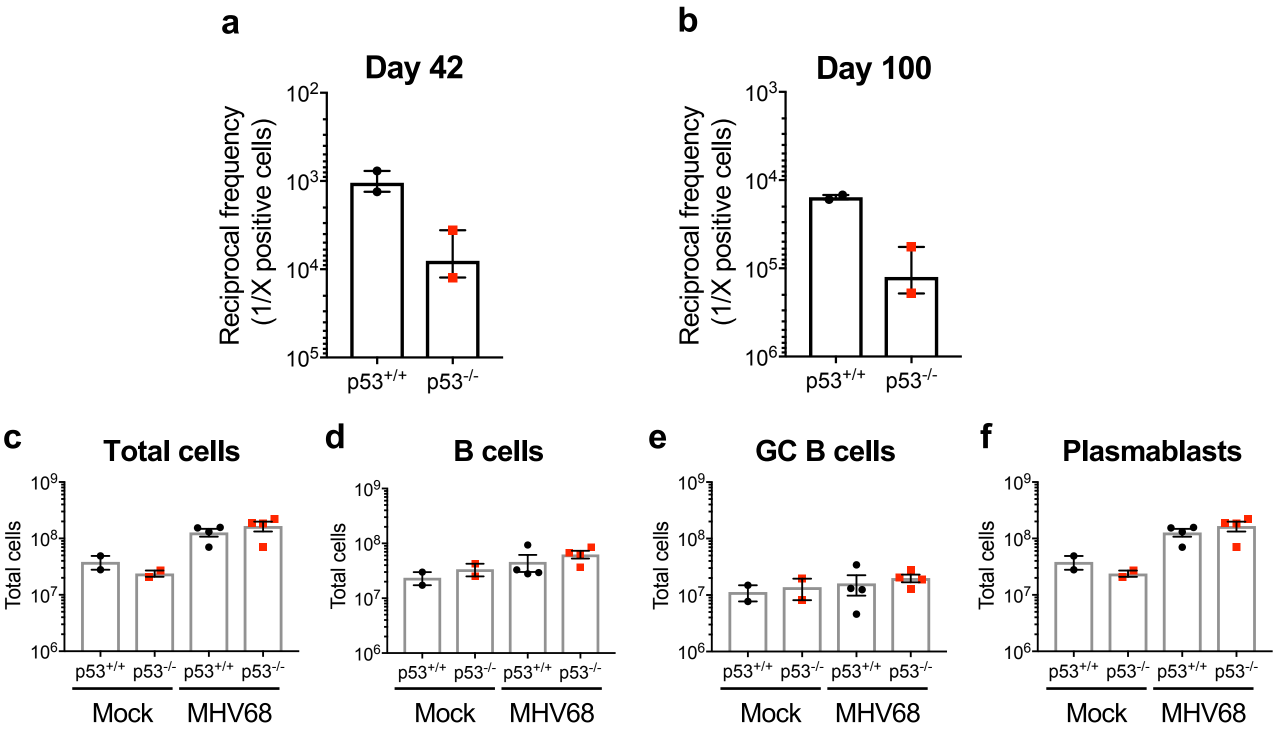

Supplementary Figure 8: Long-term MHV68 latency is reduced in p53-deficient mice. a,b, A limiting-dilution PCR analysis was performed to determine the number of splenocytes harboring MHV68 genomes on day 42 (**a**) or 100 (**b**) after infection of $p53^{+/+}$ or $p53^{-/-}$ mice with MHV68. Data represent means +/- SEM for 2 independent infections with a minimum of 3 mice per group. **c-f,** Total cells in spleens from mock-infected or MHV68-infected $p53^{+/+}$ or $p53^{-/-}$ mice were quantified and analyzed by flow cytometry. B cells were gated as $CD19^{+}/B220^{+}$, GC B cells as $GL7^{+}/CD38^{lo}$ subset of B cells, and plasmablasts as $CD138^{+}/B220^{lo}$. Data represent means +/- SEM. No significant differences were present by Mann-Whitney unpaired *t* test.

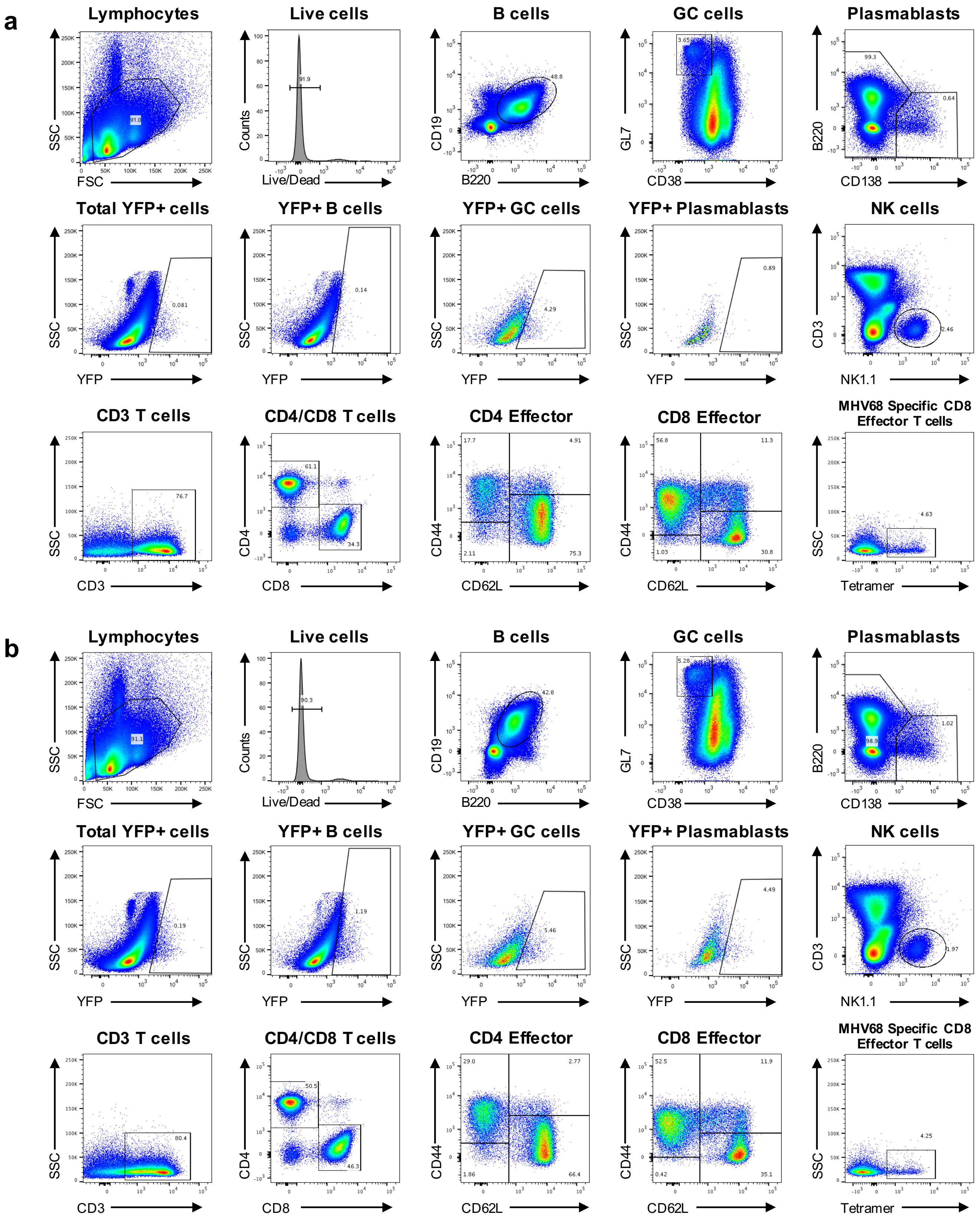

Supplementary Figure 9: Representative flow cytometry plots demonstrating gating strategies for cellular phenotyping experiments. a,b, p53$^{+/+}$ or p53$^{-/-}$ mice were infected intranasally with 10^4 PFU of H2B-YFP MHV68 and sacrificed on day 16 post-infection. Splenocytes were analyzed by flow cytometry. Specific cell types were identified as follows: B cells (CD19^{+}/B220^{+}), GC B cells (GL7^{+}/CD38^{lo}) after gating on B cells, plasmablasts (CD138^{+}/B220^{lo}), T cells (CD3^{+}) followed by CD4^{+} or CD8^{+}, effector T cells (CD62L^{-}/CD44^{+}) as CD4 or CD8 subgate, MHV68-specific effector T cells (ORF6-MHCI tet^{+}) as CD4 or CD8 effector subgate, and NK cells (CD3^{lo}/NK1.1^{+}). p53$^{+/+}$ mice are shown in **a**, and p53$^{-/-}$ mice are shown in **b**.

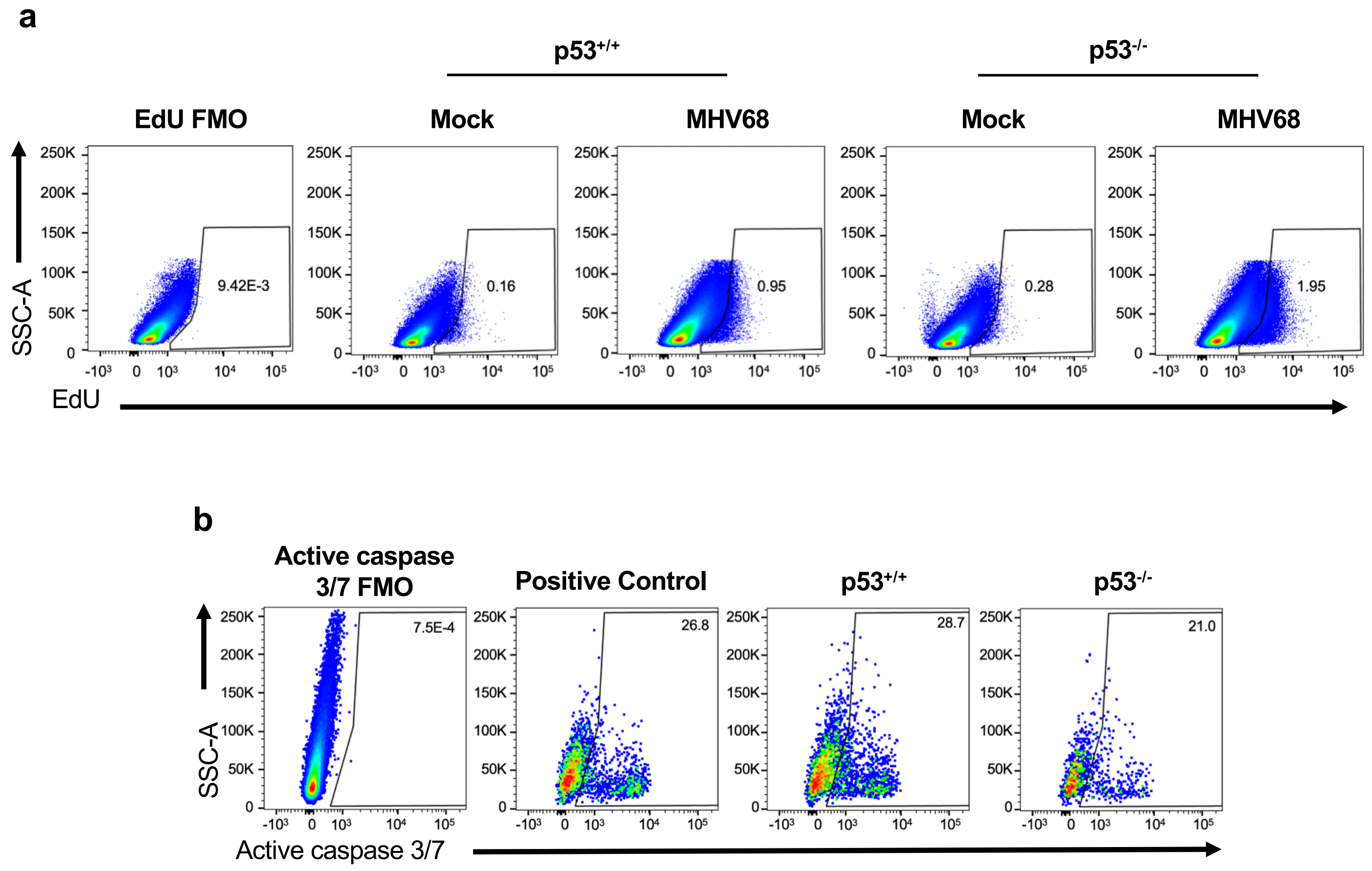

Supplementary Figure 10: Representative flow cytometry plots demonstrating gating for EdU incorporation and active caspase 3/7 assays. a,b, p53$^{+/+}$ or p53$^{-/-}$ mice were mock infected or infected intranasally with 10^4 PFU of H2B-YFP MHV68 and sacrificed on day 16 post-infection. Splenocytes were analyzed by flow cytometry. Representative flow plots from experiments described in **Figure 4a** are shown in **a**. Representative flow plots from experiments described in **Figure 4b** are shown in **b**.

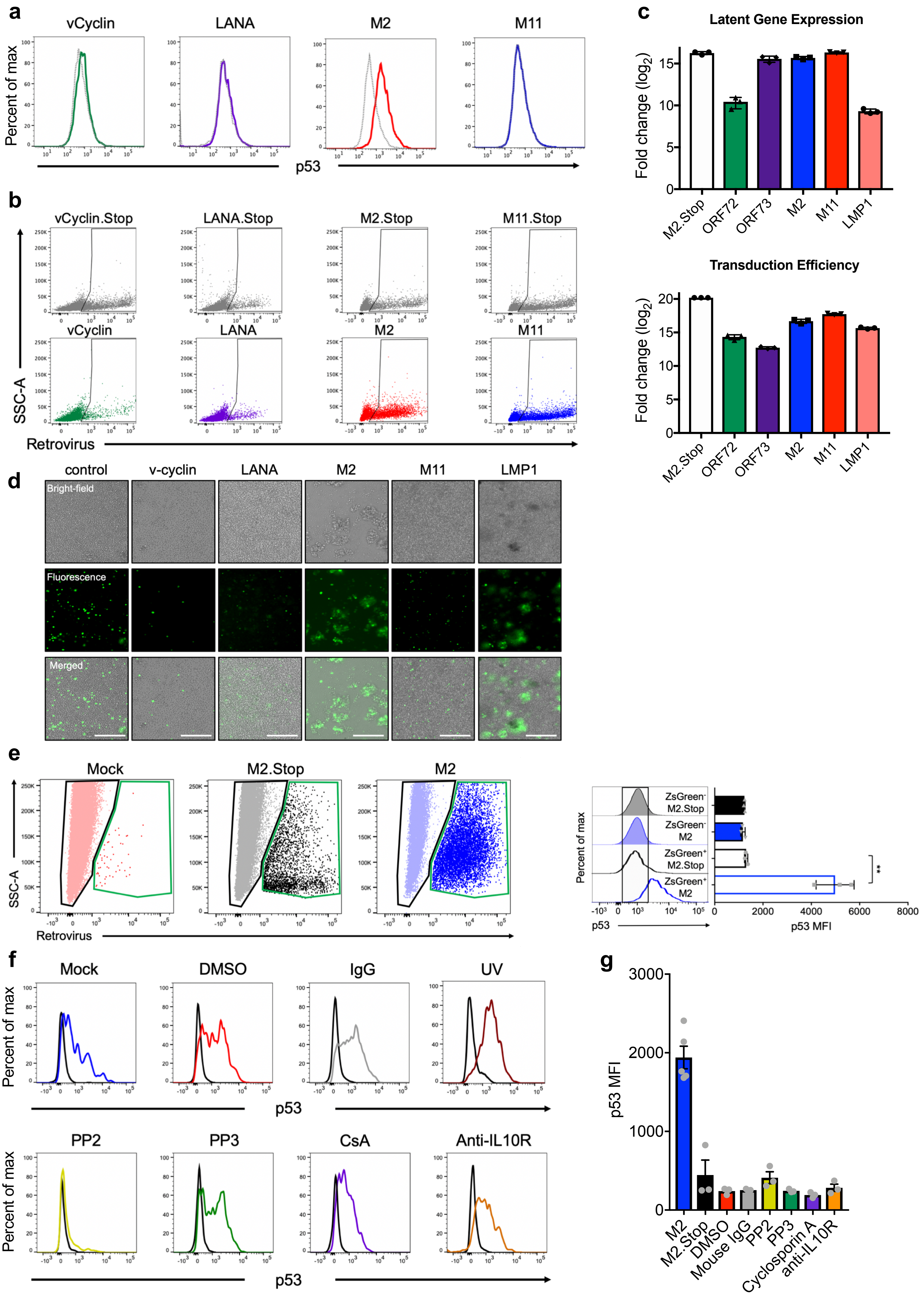
a
vCyclin
LANA
M2
M11
Percent of max
p53
b
vCyclin.Stop
LANA.Stop
M2.Stop
M11.Stop
vCyclin
LANA
M2
M11
SSC-A
Retrovirus
c
Latent Gene Expression
Fold change (log2)
M2.Stop
ORF72
ORF73
M2
M11
LMP1
Transduction Efficiency
Fold change (log2)
M2.Stop
ORF72
ORF73
M2
M11
LMP1
d
control
v-cyclin
LANA
M2
M11
LMP1
Bright-field
Fluorescence
Merged
e
Mock
M2.Stop
M2
SSC-A
Retrovirus
Percent of max
ZsGreen- M2.Stop
ZsGreen- M2
ZsGreen+ M2.Stop
ZsGreen+ M2
**
p53
p53 MFI
f
Mock
DMSO
IgG
UV
Percent of max
p53
PP2
PP3
CsA
Anti-IL10R
Percent of max
p53
g
p53 MFI
M2
M2.Stop
DMSO
Mouse IgG
PP2
PP3
Cyclosporin A
anti-IL10R

Supplementary Figure 11: Representative flow cytometry plots demonstrating gating strategies and controls for p53 expression in B cells transduced with viral latency genes. a-e, Representative flow cytometry plots and quantifications are shown for experiments described in **Figure 5**. Data in **a** show histograms in which p53 staining was overlaid for frame-shift stop control (dotted line) and viral latency gene (solid line) transduced B cells. Data in **b** show gating strategies to identify transduced cells. Data in **c** depict qRT-PCR results for latent gene expression and retroviral transduction efficiency. Latent gene expression defined as expression of the latent transcript normalized to ZsGreen expression and transduction efficiency compares ZsGreen expression normalized to naïve B cell levels. **d,** fluorescence microscopy analysis of cell morphology 72 hours after transduction of B cells with latent gene-encoding retroviruses. Scale bar indicates 250 μm. Data in **e** depict representative gating strategies for transduced and untransduced cells within single cultures and quantification of p53 staining for cells within these gates showing that p53 is induced specifically in M2-transduced B cells. of retrovirus-negative (black) and retrovirus-positive (green) populations. Data in **f** represent p53 staining in M2.Stop (black) or M2 (color) -transduced B cells in the presence of the indicated treatment. UV treatment serves as a positive control for p53 induction in non-transduced B cells. Mean fluorescence intensities of p53 staining were quantified for M2.Stop transduced B cells after the indicated treatments in **g**. M2-transduction is shown for comparison.

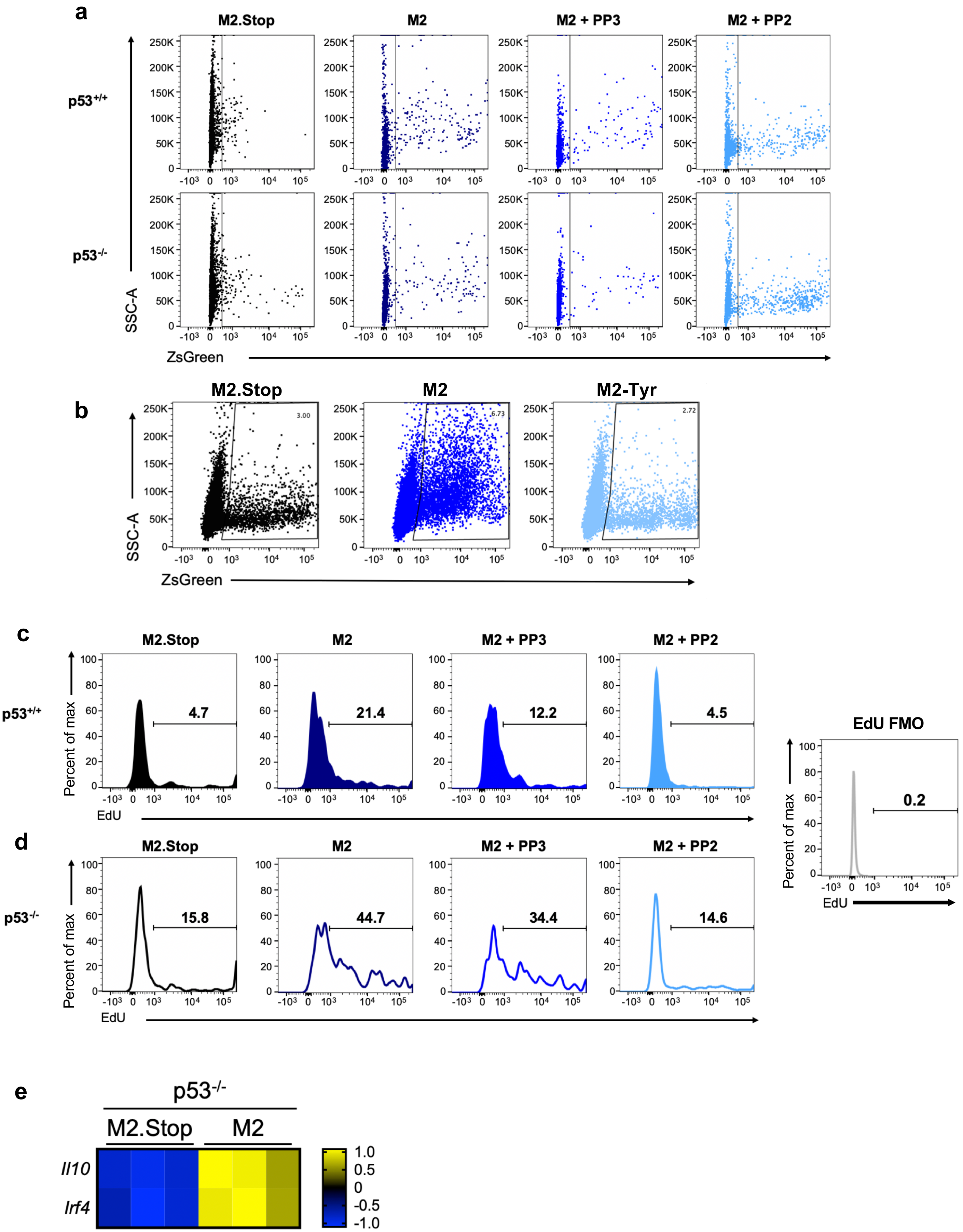
a
M2.Stop
M2
M2 + PP3
M2 + PP2
p53+/+
p53-/-
SSC-A
ZsGreen
250K
200K
150K
100K
50K
0
-10³
0
10³
10⁴
10⁵
b
M2.Stop
M2
M2-Tyr
3.00
6.73
2.72
SSC-A
ZsGreen
c
M2.Stop
M2
M2 + PP3
M2 + PP2
p53+/+
4.7
21.4
12.2
4.5
Percent of max
EdU
d
p53-/-
15.8
44.7
34.4
14.6
EdU FMO
0.2
e
p53-/-
M2.Stop
M2
Il10
Irf4
1.0
0.5
0
-0.5
-1.0

Supplementary Figure 12: Representative flow cytometry plots showing EdU incorporation and retroviral transduction. a, Flow cytometry plots showing ZsGreen detection 50 hours after transduction of $p53^{+/+}$ or $p53^{-/-}$ B cells. **b,** flow cytometry plots showing ZsGreen detection 48 hours after transduction with retroviruses encoding M2.Stop, M2, or M2-Tyr in primary B cells. **c-d,** Transduced $p53^{+/+}$ or $p53^{-/-}$ B cells were labeled with EdU for 4 hours prior to harvest and EdU detection with Click chemistry. Representative flow cytometry plots from experiments described in **Figure 7** are shown in the presence or absence of the indicated treatments. **e,** M2-associated transcript expression. Z-score heatmap comparing IL-10 and Irf4 expression in $p53^{-/-}$ primary B cells expressing M2 or M2.Stop-encoding retroviruses.

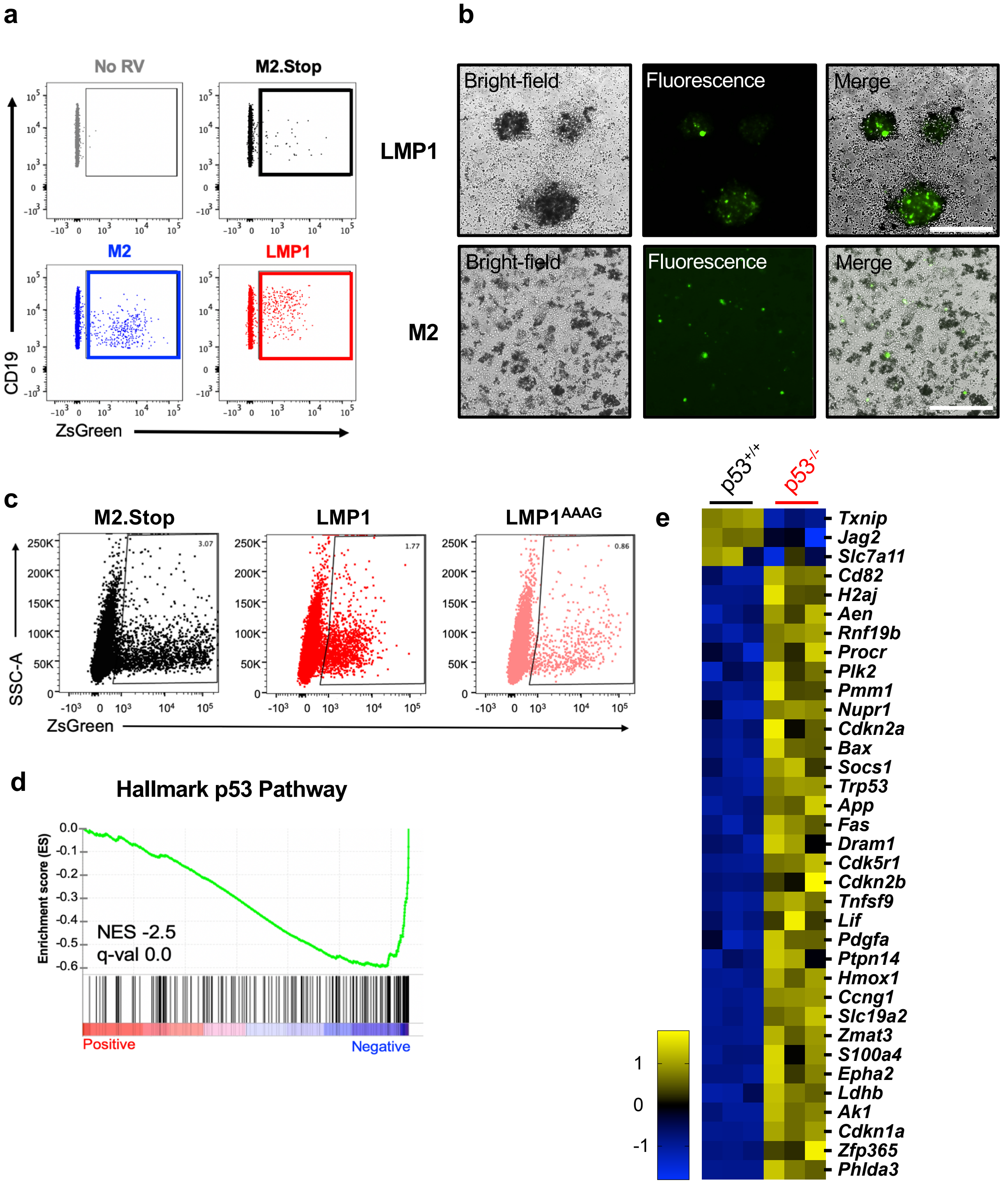

Supplementary Figure 13: Representative flow cytometry plots for defining LMP1 effects on primary mouse B cells. a, Representative flow cytometry plots showing Zs-Green detection following the indicated retroviral transductions corresponding to experiments described in **Figure 8**. **b,** fluorescence microscopy analysis showing surviving cell clusters 13 days after transduction of p53$^{-/-}$ B cells with M2 or LMP1-encoding retrovirus. Control transduction did not survive to this point. **c,** flow cytometry plots showing ZsGreen detection 48 hours after transduction with M2.Stop, LMP1, or LMP1^{AAAG} in primary B cells. **d-e,** Gene set enrichment analysis (GSEA) and hallmark gene z-score heatmaps for the hallmark p53 gene set comparing RNA-seq data from p53$^{+/+}$ and p53$^{-/-}$ B cells 4 days after transduction with LMP1 encoding retrovirus. The reference list was derived from Hallmark gene sets and compared with a pre-ranked list (by fold) of global average gene expression. Statistical scores are inset into the top right of analysis images. NES, normalized enrichment score. q-val, FDR-adjusted *p* value.

M2

$p53^{+/+}$

Rank	Top Hallmark Gene Sets	NES
1	IL-2 STAT5 Signaling	2.10
2	Epithelial mesenchymal transition	2.01
3	Angiogenesis	2.00
4	TNFA Signaling via NFKB	1.96
5	Inflammatory Response	1.94
6	p53 Pathway	1.91
1	E2F Targets	-2.05
2	G2M Checkpoint	-1.49

$p53^{-/-}$

Rank	Top Hallmark Gene Sets	NES
1	E2F Targets	3.10
2	G2M Checkpoints	2.95
3	MYC Targets	1.97
1	p53 Pathway	-2.55
2	Epithelial mesenchymal transition	-2.26
3	Angiogenesis	-2.05

LMP1

$p53^{+/+}$

Rank	Top Hallmark Gene Sets	NES
1	MYC Targets V2	2.17
2	MYC Targets V1	2.15
3	p53 Pathway	1.99
1	Protein secretion	-1.16
2	Notch Signaling	-0.99
3	Bile Acid Metabolism	-0.95

$p53^{-/-}$

Rank	Top Hallmark Gene Sets	NES
1	Interferon Alpha Response	2.12
2	Interferon Gamma Response	1.62
3	KRAS Signaling	1.40
1	p53 Pathway	-2.51
2	MYC Targets V2	-2.39
3	MYC Targets V1	-2.35

Supplementary Figure 14: Top hallmark gene sets identified in GSEA for M2 and LMP1 expressing B cells. The tables show significantly up-regulated (yellow) or down-regulated (blue) hallmark gene sets for the indicated comparisons. The reference list was derived from Hallmark gene sets and compared with a pre-ranked list (by fold) of global average gene expression. NES, normalized enrichment score.
