# Supplementary material for: Intrinsic p53 activation restricts gammaherpesvirus driven germinal center B cell expansion during latency establishment
Source: Nat Commun. 2025 Jan 22;16:951. doi: 10.1038/s41467-025-56247-5 (PMC11754798; doi:10.1038/s41467-025-56247-5)
Supplement: Supplementary file 2 — Reporting Summary [file 41467_2025_56247_MOESM2_ESM.pdf]

nature portfolio

Corresponding author(s): J. Craig Forrest
Last updated by author(s): Dec 20, 2024

Reporting Summary

Nature Portfolio wishes to improve the reproducibility of the work that we publish. This form provides structure for consistency and transparency in reporting. For further information on Nature Portfolio policies, see our Editorial Policies and the Editorial Policy Checklist.

Statistics

For all statistical analyses, confirm that the following items are present in the figure legend, table legend, main text, or Methods section.

n/a	Confirmed	
☐	☒	The exact sample size (*n*) for each experimental group/condition, given as a discrete number and unit of measurement
☐	☒	A statement on whether measurements were taken from distinct samples or whether the same sample was measured repeatedly
☐	☒	The statistical test(s) used AND whether they are one- or two-sided<br>*Only common tests should be described solely by name; describe more complex techniques in the Methods section.*
☐	☒	A description of all covariates tested
☐	☒	A description of any assumptions or corrections, such as tests of normality and adjustment for multiple comparisons
☐	☒	A full description of the statistical parameters including central tendency (e.g. means) or other basic estimates (e.g. regression coefficient) AND variation (e.g. standard deviation) or associated estimates of uncertainty (e.g. confidence intervals)
☐	☒	For null hypothesis testing, the test statistic (e.g. *F*, *t*, *r*) with confidence intervals, effect sizes, degrees of freedom and *P* value noted<br>*Give P values as exact values whenever suitable.*
☒	☐	For Bayesian analysis, information on the choice of priors and Markov chain Monte Carlo settings
☒	☐	For hierarchical and complex designs, identification of the appropriate level for tests and full reporting of outcomes
☒	☐	Estimates of effect sizes (e.g. Cohen's *d*, Pearson's *r*), indicating how they were calculated

Our web collection on statistics for biologists contains articles on many of the points above.

Software and code

Policy information about availability of computer code

Data collection	The flow data were collected using an LSRFortessa (Becton Dickinson). Ultra-low input RNAseq samples were sequenced on the Illumina HiSeq® 2500 with a 1x100 bp single-end configuration (GeneWiz). Standard RNA-seq sequencing was performed on a DNBSEQ-G400 (BGI Genomics Co).
Data analysis	All data was analyzed using GraphPad Prism software (GraphPad Software, http://www.graphpad.com, La Jolla, CA). PCR analyzed using GeneGlobe RT2 Profiler PCR Data Analysis software. Flow data was analyzed using FlowJo (10.4.2) software. RNA-Seq - Raw sequence reads were quality-checked using fastQC and trimmed based on quality (Phred >30). After trimming, reads <30 bp in size were discarded. Trimmed sequence reads were mapped to reference genome mm10 using STAR aligner with default parameters. The gene count tables were extracted from alignment results using bedtool2 software. Read counts were normalized using Voom method. Normalized read counts were analyzed using DEseq2.Representative gene set enrichment analysis (GSEA) was performed using the reference list derived from Hallmark gene sets and compared with a pre-ranked list (by fold) of global average gene expression.

For manuscripts utilizing custom algorithms or software that are central to the research but not yet described in published literature, software must be made available to editors and reviewers. We strongly encourage code deposition in a community repository (e.g. GitHub). See the Nature Portfolio guidelines for submitting code & software for further information.

Data

Policy information about availability of data

All manuscripts must include a data availability statement. This statement should provide the following information, where applicable:

- Accession codes, unique identifiers, or web links for publicly available datasets
- A description of any restrictions on data availability
- For clinical datasets or third party data, please ensure that the statement adheres to our policy

mRNAseq data are deposited under GEO Accession # GSE225579 (Reviewer token: wnwdoamcrbupnat). (https://www.ncbi.nlm.nih.gov/geo/query/acc.cgi?acc=GSE225579).

Research involving human participants, their data, or biological material

Policy information about studies with human participants or human data. See also policy information about sex, gender (identity/presentation), and sexual orientation and race, ethnicity and racism.

Reporting on sex and gender	N/A
Reporting on race, ethnicity, or other socially relevant groupings	N/A
Population characteristics	N/A
Recruitment	N/A
Ethics oversight	N/A

Note that full information on the approval of the study protocol must also be provided in the manuscript.

Field-specific reporting

Please select the one below that is the best fit for your research. If you are not sure, read the appropriate sections before making your selection.

☒ Life sciences ☐ Behavioural & social sciences ☐ Ecological, evolutionary & environmental sciences

For a reference copy of the document with all sections, see nature.com/documents/nr-reporting-summary-flat.pdf

Life sciences study design

All studies must disclose on these points even when the disclosure is negative.

Sample size	Let SD be the within-group std dev of Y (outcome of interest). With n=3 pools (3-5 mice per pool), we will have about .8 power to detect a difference in means of size 3.1 SD using a two-sample, .05 level t-test. Mean fluorescence intensity at each timepoint will be determined and evaluated for statistical significance using a .05 level two-tailed student's t-test. With 6 animals per mouse-virus-timepoint group, we will have at least .80 power for detecting a difference of size 1.33 standard deviations using a .05 level t-test for each comparison of interest. This calculation does not control for multiple comparisons.
Data exclusions	N/A
Replication	Data represent at least biological replicates of 3-5 mice per pool.
Randomization	For every experiment, experimental controls were run in parallel with mock controls. For animal groups, cages were picked randomly based on the number of animals following breeding and genotyping.
Blinding	Investigators were not blinded during sample collection. However, all experiments were performed in biological replicates across years of experiments.

Reporting for specific materials, systems and methods

We require information from authors about some types of materials, experimental systems and methods used in many studies. Here, indicate whether each material, system or method listed is relevant to your study. If you are not sure if a list item applies to your research, read the appropriate section before selecting a response.

Materials & experimental systems

n/a	Involved in the study
☐	☒ Antibodies
☐	☒ Eukaryotic cell lines
☒	☐ Palaeontology and archaeology
☐	☒ Animals and other organisms
☒	☐ Clinical data
☒	☐ Dual use research of concern
☒	☐ Plants

Methods

n/a	Involved in the study
☒	☐ ChIP-seq
☐	☒ Flow cytometry
☒	☐ MRI-based neuroimaging

Antibodies

Antibodies used

P53 (1C12) Mouse mAb (AF647) Cell Signaling 2533S
GFP Antibody – Goat Polyclonal Rockland 600-401-215
Anti-mouse CD38 (PE-Cy7) BioLegend 102718
Anti-mouse CD138 (PE) BioLegend 142504
Anti-mouse CD19 (BV650) Biolegend 115541
Anti-mouse GL7 (EF660) eBio 50-5902-82
eBioscience Fixable Viability Dye (eF780) eBio 65-0865-18
Anti-mouse CD45R/B220 (rF710) Tonbo 80-0452-U100
PcP-Cy5.5 Anti-Human/Mouse CD11b Tonbo 65-0112-U100
PcP-Cy5.5 Anti-Mouse CD11c Tonbo 65-0114-U100
PcP-Cy5.5 Anti-Mouse TER-119 Tonbo 65-5921-U100
PcP-Cy5.5 Anti-Mouse CD45 Tonbo 65-0452-U100
VF450 CD8 Tonbo 75-0081-U100
redFluor710/AF700 CD4 Tonbo 80-0042-U100
PcP-Cy5.5 Anti-Mouse CD3 Tonbo 65-0031-U100
PE anti-mouse/human CD44 Antibody Biolegend 103008
Brilliant Violet 510™ anti-mouse CD3ε Antibody Biolegend 100353
Brilliant Violet 605™ anti-mouse CD62L Antibody Biolegend 104438
Anti-Mouse NK1.1 (PK136) Biolegend 108701
Anti-Goat AF488 Invitrogen A-11055
Anti-mouse IL-10R (CD210) BioXCell BP0050
Mouse IgG Isotype Control ThermoFisher 31903
Donkey anti-rat AF647-conjugate Jackson ImmunoResearch 712-605-150
donkey anti-goat Alexa Fluor 488-conjugate Jackson ImmunoResearch 705-545-147
Purified anti-mouse/human CD45R/B220 Antibody Biolegend 103201

Validation

P53 (1C12) Mouse mAb (AF647) Cell Signaling 2533S https://www.cellsignal.com/products/antibody-conjugates/p53-1c12-mouse-mab-alexa-fluor-647-conjugate/2533?site-search-type=Products&N=4294960176%204294956287&Ntt=p53&fromPage=plp
GFP Antibody – Goat Polyclonal Rockland 600-401-215 https://www.rockland.com/categories/primary-antibodies/gfp-antibody-600-101-215/
Anti-mouse CD38 (PE-Cy7) BioLegend 102718 https://www.biolegend.com/en-us/products/pe-cyanine7-anti-mouse-cd38-antibody-3926
Anti-mouse CD138 (PE) BioLegend 142504 https://www.biolegend.com/en-us/products/pe-anti-mouse-cd138-syndecan-1-antibody-7519
Anti-mouse CD19 (BV650) Biolegend 115541 https://www.biolegend.com/en-us/products/brilliant-violet-650-anti-mouse-cd19-antibody-7851
Anti-mouse GL7 (EF660) eBio 50-5902-82 https://www.thermofisher.com/antibody/product/GL7-Antibody-clone-GL-7-GL7-Monoclonal/50-5902-82
eBioscience Fixable Viability Dye (eF780) eBio 65-0865-18 https://www.thermofisher.com/order/catalog/product/65-0865-14
Anti-mouse CD45R/B220 (rF710) Tonbo 80-0452-U100 https://cytekbio.com/products/redfluor-710-anti-human-mouse-cd45r-b220-ra3-6b2?variant=40581186322468
PcP-Cy5.5 Anti-Human/Mouse CD11b Tonbo 65-0112-U100 https://cytekbio.com/products/percp-cyanine5-5-anti-human-mouse-cd11b-m1-70?variant=40581197168676
PcP-Cy5.5 Anti-Mouse CD11c Tonbo 65-0114-U100
PcP-Cy5.5 Anti-Mouse TER-119 Tonbo 65-5921-U100 https://cytekbio.com/products/percp-cyanine5-5-anti-mouse-ter-119-ter-119?variant=40581194055716
PcP-Cy5.5 Anti-Mouse CD45 Tonbo 65-0452-U100 https://cytekbio.com/products/percp-cyanine5-5-anti-human-mouse-cd45r-b220-ra3-6b2
VF450 CD8 Tonbo 75-0081-U100 https://cytekbio.com/products/violetfluor-450-anti-mouse-cd8a-53-6-7?variant=40581181472804
redFluor710/AF700 CD4 Tonbo 80-0042-U100 https://cytekbio.com/products/redfluor-710-anti-mouse-cd4-rm4-5?variant=40581185568804
PcP-Cy5.5 Anti-Mouse CD3 Tonbo 65-0031-U100 https://cytekbio.com/products/percp-cyanine5-5-anti-mouse-cd3e-145-2c11?variant=40581195268132
PE anti-mouse/human CD44 Antibody Biolegend 103008 https://www.biolegend.com/en-us/products/pe-anti-mouse-human-cd44-antibody-2206
Brilliant Violet 510™ anti-mouse CD3ε Antibody Biolegend 100353 https://www.biolegend.com/en-us/products/brilliant-violet-510-anti-mouse-cd3epsilon-antibody-11973
Brilliant Violet 605™ anti-mouse CD62L Antibody Biolegend 104438 https://www.biolegend.com/en-us/products/brilliant-violet-605-

anti-mouse-cd62l-antibody-7687
Anti-Mouse NK1.1 (PK136) Biolegend 108701 https://www.biolegend.com/en-us/products/purified-anti-mouse-nk-1-1-antibody-432
Anti-Goat AF488 Invitrogen A-11055 https://www.thermofisher.com/antibody/product/Donkey-anti-Goat-IgG-H-L-Cross-Adsorbed-Secondary-Antibody-Polyclonal/A-11055
Anti-mouse IL-10R (CD210) BioXCell BP0050 https://bioxcell.com/invivoplus-anti-mouse-il-10r-cd210-bp0050
Mouse IgG Isotype Control ThermoFisher 31903 https://www.thermofisher.com/antibody/product/Mouse-IgG-Polyclonal/31903
Donkey anti-rat AF647-conjugate Jackson ImmunoResearch 712-605-150 https://www.jacksonimmuno.com/catalog/products/712-605-150
donkey anti-goat Alexa Fluor 488-conjugate Jackson ImmunoResearch 705-545-147 https://www.jacksonimmuno.com/catalog/products/705-545-147
Purified anti-mouse/human CD45R/B220 Antibody
Biolegend 103201
https://www.biolegend.com/en-us/products/purified-anti-mouse-human-cd45r-b220-antibody-449

Eukaryotic cell lines

Policy information about cell lines and Sex and Gender in Research

Cell line source(s)

Murine embryonic fibroblasts Forrest Lab - pool from un-sexed embryos
Phoenix-ECO ATCC CRL-3214
Primary B cells - pooled from male and female

Authentication

None of the cells were authenticated. Primary B cell enrichment was confirmed after AutoMac separation by flow cytometry.

Mycoplasma contamination

Cells were free of mycoplasma contamination. Tested by PCR (ABM, cat# G238)

Commonly misidentified lines
(See ICLAC register)

Name any commonly misidentified cell lines used in the study and provide a rationale for their use.

Animals and other research organisms

Policy information about studies involving animals; ARRIVE guidelines recommended for reporting animal research, and Sex and Gender in Research

Laboratory animals

C57BL/6J Jackson Laboratory 000664
B6.129S2-Trp53tm1Tyj/J Jackson Laboratory 002101
B6.Cg-Gt(ROSA)26Sortm6(CAG-ZsGreen1)Hze/J Jackson Laboratory 007906
B6.Cg-Gt(ROSA)26Sortm14(CAG-tdTomato)Hze/J Jackson Laboratory 007914
B6.129P2-Aicdatm1(cre)Mnz/J Jackson Laboratory 007770
B6.129S2-Ifnar1tm1Agt/Mmjax Jackson Laboratory 032045-JAX

Wild animals

N/A

Reporting on sex

Both male and female mice are used for these experiments, and no overt differences in phenotypes due to sex are known to exist.

Field-collected samples

N/A

Ethics oversight

All mice were housed and cared for according to the guidelines of UAMS Department of Laboratory Animal Medicine and all state and federal requirements. At UAMS, mice are housed in IVC cages in a room with a 12:12 light cycle and environmental conditions of 72F and humidity of 30-70%.

Note that full information on the approval of the study protocol must also be provided in the manuscript.

Plants

Seed stocks

N/A

Novel plant genotypes

N/A

Authentication

N/A

Flow Cytometry

Plots

Confirm that:

☒ The axis labels state the marker and fluorochrome used (e.g. CD4-FITC).

☒ The axis scales are clearly visible. Include numbers along axes only for bottom left plot of group (a 'group' is an analysis of identical markers).

☒ All plots are contour plots with outliers or pseudocolor plots.

☒ A numerical value for number of cells or percentage (with statistics) is provided.

Methodology

Sample preparation	Spleens were homogenized in a tenBroek tissue disrupter. Red blood cells were lysed by incubating tissue homogenate in 8.3 g/L ammonium chloride for 10 minutes at room temperature with shaking. Cells were filtered through a 40-micron mesh to reduce clumping. Cells were washed with FACS buffer (0.2% BSA, 1 mM in PBS) before blocking with Fc block (Invitrogen) and incubation with eF780 live/dead viability stain (eBioscience) for 10 minutes at 4ºC. Surface staining was then performed with antibodies diluted at 1:300 for 30 minutes incubation time at 4ºC. For intracellular stains, cells were fixed and permeabilized using a FoxP3 staining kit (eBioscience) following the manufacturer's guidelines.
Instrument	The data were collected using an LSRFortessa (Becton Dickinson). For cell sorting - immediately subjected to fluorescent-activated cell sorting (FACS) performed on a FACS Aria flow cytometer (BD Biosciences).
Software	Data was analyzed using FlowJo (10.4.2) software.
Cell population abundance	Cell populations from sorted cells are shown in Figure 6 and Supplementary Fig. 2.
Gating strategy	Gating strategies are noted in the primary figures or supplementary figures. Figure 1A, 6A. Supplementary Fig 1a, 2a,7b-d, 9, 10, 11b & e, 12, 13a & c.

☒ Tick this box to confirm that a figure exemplifying the gating strategy is provided in the Supplementary Information.
